# Polyacylated Anthocyanins in Bluish-Purple Petals of Chinese Bellflower, *Platycodon grandiflorum*

**DOI:** 10.3390/ijms22084044

**Published:** 2021-04-14

**Authors:** Tadao Kondo, Seiji Hagihara, Yoshiaki Takaya, Kumi Yoshida

**Affiliations:** 1 Graduate School of Informatics, Nagoya University, Chikusa, Nagoya 464-8601, Japan; kondot@info.human.nagoya-u.ac.jp (T.K.); hagihara.seiji@j.mbox.nagoya-u.ac.jp (S.H.); 2 Faculty of Pharmacy, Meijo University, 150 Yagotoyama, Tenpaku, Nagoya 468-8503, Japan; ytakaya@meijo-u.ac.jp

**Keywords:** bluish-purple, delphinidin, intramolecular stacking, *Platycodon grandiflorum*, polyacylated anthocyanin, stability

## Abstract

The bluish-purple petals of Chinese bellflower, *Platycodon grandiflorum* (kikyo in Japanese), contain platyconin (**1**) as the major anthocyanin. Platyconin (**1**) is a polyacylated anthocyanin with two caffeoyl residues at the 7-position, and its color is stable in a diluted, weakly acidic aqueous solutions. HPLC analysis of the fresh petal extract showed the presence of several minor pigments. Photo-diode array detection of minor pigments suggested that some of these were polyacylated anthocyanins. To establish the relationship between structure and stability of the acylated anthocyanins and to obtain information on their biosynthetic pathways, minor pigments were isolated from the petals, and their structures were determined by MS and NMR analyses. Four known (**2**–**5**) and three new anthocyanins (**6**–**8**) were identified, which contained a delphinidin chromophore, and four of these (**5**–**8**) were diacylated anthocyanins, in which the acyl-glucosyl-acyl-glucosyl chain was attached at the 7-*O*-position of the delphinidin chromophore. These diacylated anthocyanins exhibited a bluish-purple color at pH 6, which was stable for more than a week.

## 1. Introduction

Many flower petals with blue to bluish-purple colors contain unique anthocyanins known as polyacylated anthocyanins, which contain two or more aromatic acyl residues, such as *p*-coumaroyl, caffeoyl, feruloyl, sinapoyl, and *p*-hydroxybenzoyl moieties [1,2,3,4,5]. Polyacylated anthocyanins are stable in dilute, weakly acidic to neutral aqueous solutions (<50 μM) [1,2,5]. Although simple anthocyanins substituted only with sugars rapidly decompose to provide a colorless pseudobase in weakly acidic to neutral conditions, polyacylated anthocyanins are present in their colored forms for a few hours to months [1,4,6]. This is because the intramolecular stacking of the aromatic acyl moieties to the anthocyanidin chromophore via hydrophobic interaction prevents these from becoming colorless [1,2,5].

**Figure 1:**
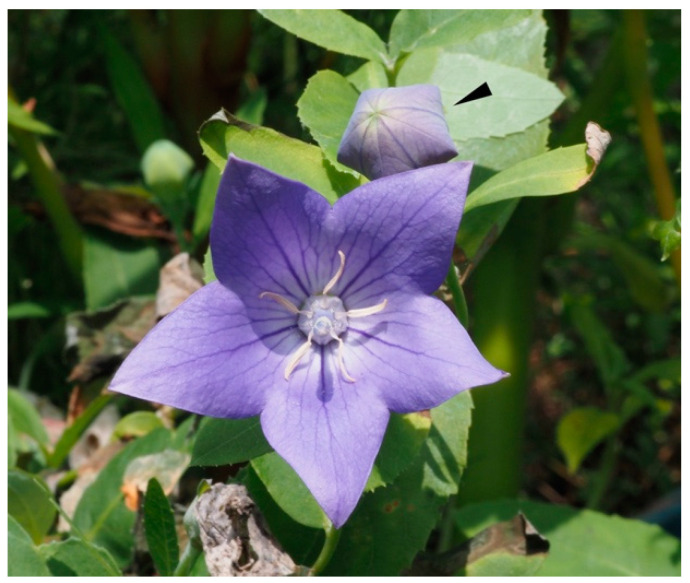
Flower (center) and bud (indicated with a black arrow) of *P. grandiflorum* (kikyo in Japanese).

Platyconin (**1**) in the bluish-purple petals of Chinese bellflower, *Platycodon grandiflorum* (kikyo in Japanese, Figure 1), was the first reported polyacylated anthocyanin in 1971 [6]. The complete structure of **1** was determined by Goto et al. in 1983 [7]. It contains a delphinidin chromophore, which is substituted by a rutinosyl residue at the 3-position, and a glucosyl-caffeoyl-glucosyl-caffeoyl-glucosyl chain at the 7-*O*-position of the chromophore [7]. To date, many polyacylated anthocyanins with more than hundreds of structural variations have been reported [4]; however, pigments with an acyl substituted chain only at the 7-OH position of the chromophore are limited [3] and found in Campanulaceae [6,7,8], Ranunculaceae [9,10], and Goodeniaceae families of plants [11]. For chemotaxonomical studies and understanding the biosynthetic pathways of these polyacetylated anthocyanins, a survey of structurally similar pigments to **1**, together with their isolation and unambiguous structure elucidation is essential. Therefore, the anthocyanins present in the petals of Chinese bellflower were investigated. Employing large-scale extraction followed by purification with repeated chromatography, six pure pigments were obtained in addition to platyconin (**1**). Their structures were elucidated by a combination of instrumental analysis (MS and NMR) and chemical degradation. Herein, the structures of four known (**2**–**5**) and three new anthocyanins (**6**–**8**) with their stability data and chemical characteristics are described.

## 2. Results and Discussion

### 2.1. HPLC Analysis of Minor Anthocyanins

**Figure 2:**
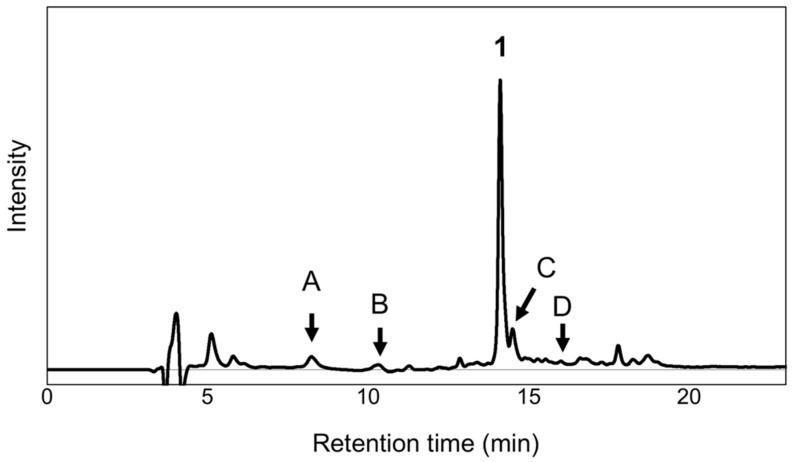
HPLC chromatogram of the petal extracts of *P. grandiflorum* (detection: 280 nm).

The extract of the fully opened flower petals of *P. grandiflorum* was analyzed using HPLC with photodiode array detection. The chromatogram of the extract showed the presence of several minor peaks attributable to the anthocyanins (Figure 2). The peak with the highest intensity was identified as platyconin (**1**) by comparison with the authentic pigment. Other peaks including those of the polyacylated anthocyanins were analyzed based on the UV spectra obtained by photodiode array detection. Peaks A and B corresponded to simple anthocyanins without any aromatic acyl moieties. Peaks C and D were correlated to the diacylated anthocyanins because the ratio of absorbance at λuv_max_ and λvis_max_ was approximately 1 (Figure S1), similar to that of 1 [5]. After purification, it was confirmed that peak A contained **2**, peak B constituted two simple anthocyanins (**3** and **4**), peak C comprised three diacylated anthocyanins (**5**–**7**) with similar structures, and peak D contained **8**.

### 2.2. Purification of Anthocyanins

After preliminary experiments, it was determined that no malonyl ester and no other labile partial structures were present in the anthocyanins of the petals. Therefore, methanol (MeOH) was used for extraction and crude purification. Frozen petals were extracted using aq. HCl-MeOH and the extract was evaporated and washed with ethyl ether (Et_2_O). The aqueous solution was purified using Amberlite XAD-7 column chromatography. From 1.2 kg of petals, 1.8 g of crude anthocyanin fraction was obtained. The crude fraction was further purified using preparative ODS HPLC and recycled HPLC-elution was performed to afford pure **1** and **3**–**8** as dark-red trifluoroacetic acid (TFA) salts.

### 2.3. Alkaline Hydrolysis of Platyconin *(**1**)*

HPLC analysis of peak A indicated that it constitutes a simple anthocyanin without any aromatic acyl moieties, and this was identified as bisdeacylplatyconin (**2**) based on a previous report [7]. To further confirm its identity, alkaline hydrolysis of **1** was carried out using aq. NaOH in CH_3_OH under argon atmosphere. After purification using preparative HPLC, pure **2** was obtained as a dark-red TFA salt.

### 2.4. Structure Elucidation of Anthocyanins

High resolution mass spectrometry (HR-MS) analyses of **1** and **5**–**8** afforded their molecular weights, which are listed in Table 1 (Figures S2–S5). The ^1^H NMR spectra of **3**–**8** indicated the presence of the delphinidin chromophore in all compounds (Figures S6–S12). Furthermore, data analysis suggested that **3** and **4** did not have any aromatic acyl residue, **5** lacked a rhamnosyl residue, **6** and **7** lacked one oxygen atom, and **8** lacked one glucosyl residue in comparison to **1**.

**Scheme 1:**
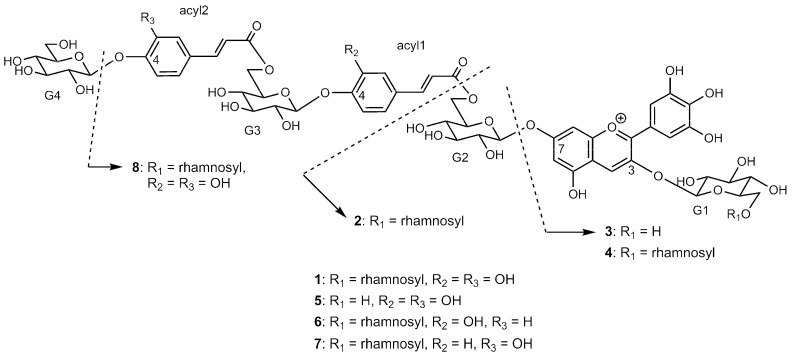
Structures of petal anthocyanins **1**–**8** in *platycodon grandiflorum*.

Various 1D and 2D NMR experiments allowed the unambiguous assignment of all signals (Table 2, Figures S6–S13). For structure elucidation, the analyses were performed according to our general procedure [12,13,14]. All sugar signals were assigned using 1D and 2D TOCSY NMR data, while the positions at which the sugars were connected were determined using nuclear Overhauser effect (NOE), and the connectivity of the acyl moiety was determined via HMBC correlations. After determining the identity and positions of the acyl moiety and sugar residue, the structures of **5**–**8** were elucidated, as shown in Scheme 1. The structure of **5** was identified as 3-*O*-*β*-d-glucopyranosyl-7-*O*-(6-*O*-(4-*O*-(6-*O*-(4-*O*-*β*-d-glucopyranosyl-(*E*)-caffeoyl)-*β*-d-glucopyranosyl)-(*E*)-caffeoyl)-*β*-d-glucopyranosyl)delphinidin, which has been reported as a derivative obtained by the acid treatment of Leschenaultia petal anthocyanin [11]. The structures of new diacylated anthocyanins **6**–**8** were identified as 3-*O*-(6-*O*-*α*-l-rhamnopyranosyl-*β*-d-glucopyranosyl)-7-*O*-(6-*O*-(4-*O*-(6-*O*-(4-*O*-*β*-d-glucopyranosyl-(*E*)-*p*-coumaroyl)-*β*-d-glucopyranosyl)-(*E*)-caffeoyl)-*β*-d-glucopyranosyl)delphinidin, 3-*O*-(6-*O*-*α*-l-rhamnopyranosyl-*β*-d-glucopyranosyl)-7-*O*-(6-*O*-(4-*O*-(6-*O*-(4-*O*-*β*-d-glucopyranosyl-(*E*)-caffeoyl)-*β*-d-glucopyranosyl)-(*E*)-*p*-coumaroyl)-*β*-d-glucopyranosyl)delphinidin, and 3-*O*-(6-*O*-*α*-l-rhamnopyranosyl-*β*-d-glucopyranosyl)-7-*O*-(6-*O*-(4-*O*-(6-*O*-(*E*)-caffeoyl-*β*-d-glucopyranosyl)-(*E*)-caffeoyl)-*β*-d-glucopyranosyl)delphinidin, respectively.

Anthocyanin **2** was identified as 3-*O*-(6-*O*-*α*-L-rhamnopyranosyl-*β*-d-glucopyranosyl)-7-*O*-β-d-glucopyranosyldelphinidin by comparison with an authentic sample that was obtained by the alkaline hydrolysis of **1** [7]. The structure of **3** and **4** were identified as 3-*O*-*β*-d-glucopyranosyldelphinidin and 3-*O*-(6-*O*-*α*-L-rhamnopyranosyl-*β*-d-glucopyranosyl)delphinidin, respectively, based on NMR analysis and comparison with authentic samples.

These results indicated that the acylation position was the same as 7-OH of the delphinidin chromophore in all the polyacylated pigments; however, the acyl species varied. Two of the new diacylated anthocyanins contained one caffeoyl and one *p*–coumaroyl residue, but the acyl groups were connected in different orders. This suggested that the biosynthetic route of acylation in these polyacylated anthocyanins may not be strictly controlled, and was redundant. The acyl chain at the 7-position in polyacylated anthocyanin found in the violet and blue petals of delphinium was reported to be extended in vacuoles using a bi-functional substrate, acylglucose [15,16]. The minor pigments found in the petals of *P. grandiflorum* may also be biosynthesized via a similar route by employing the bi-functional substrates, caffeoyl-glucose and *p*-coumaroyl glucose. For further studies, a survey of these substrate candidates and cloning of glucosyltransferase and acyltransferase are required.

### 2.5. Color and Stability of Chinese Bellflower Anthocyanins

**Figure 3:**
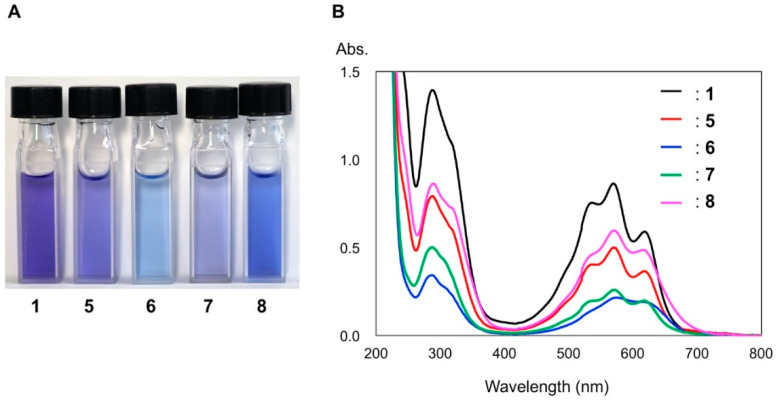
Colors and UV-Vis spectra of diacylated anthocyanins **1** and **5**–**8** (50 μM in 200 mM phosphate buffer solutions at pH 6.0, cell length: 10 mm). (**A**): Photograph of the solutions of **1** and **5**–**8**. (**B**): UV-Vis spectra of **1** and **5**–**8**.

To investigate the color development and stability of anthocyanins in Chinese bellflower petals, each pigment isolated from the petals was dissolved in an aqueous buffered solution at pH 6.0, and the UV-Vis spectra were recorded (Figure 3). The five pigments had similar structures; however, the hue of each solution was different (Figure 3A). Among these pigments, platyconin (**1**) was violet, and **6** and **8** showed a bluish color in comparison to **1**. Interestingly, the structural difference between **6** and **7** was only the order of the caffeoyl and *p*-coumaroyl moieties at the 7-OH position of the delphinidin chromophore, but these two compounds showed different hues. All diacylated anthocyanins showed three λ_max_ values at 617, 570, and 536 nm in the visible region (Figure 3B). However, the absorbance at λ_max_ was different in each pigment. For example, the ratios of the absorbances at 617 nm to 570 nm in **1** was 0.68, and those of **5**–**8** were 0.73, 0.89, 0.76, and 0.82, respectively. The difference in hue was attributed to these variations in the absorbances; **6** and **8** showed a bluish hue, while **1**, **5**, and **7** showed a violet color. These differences may be due to the differences in the p*K*a values, which are affected by the modification of acylation and glycosylation.

**Figure 4:**
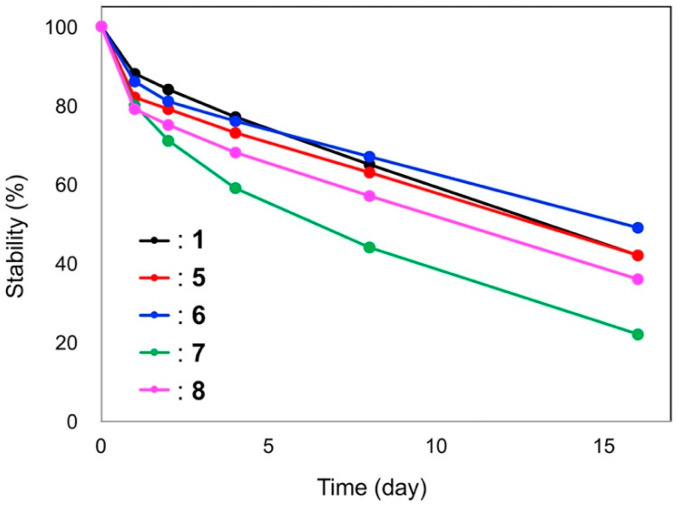
Color stability of anthocyanins **1** and **5**–**8** (50 μM in 200 mM phosphate buffer solutions at pH 6.0, cell length: 10 mm). Each solution was stored in the dark at room temperature and the ratio of the absorbance at λvis_max_ was plotted.

The color stability in a diluted aqueous solution at pH 6 was monitored by placing the solution in the dark at room temperature. As shown in Figure 4, all diacylated anthocyanins (**1**, **5**–**8**) are stable, and in contrast, the three non-acylated anthocyanins (**2**–**4**) are unstable and rapidly decolorize within a few minutes, which agrees with a previous report [7]. Among the diacylated anthocyanins, **1**, **5**, and **6** showed similar stabilities, i.e., >60% of the color was retained after storage for one weak. Compound **8** was less stable compared to **1**, **5**, and **6** (nearly 50% color was retained after one week), and **7** was the most unstable diacylated pigment. Interestingly, **6** and **7** showed different stabilities, and only the order of caffeoyl and *p*-coumaroyl substitution was different in these compounds. The high stability of the diacylated anthocyanins (**1**, **5**–**8**) could be attributed due to the intramolecular stacking of the aromatic acyl residues to the anthocyanidin chromophore [1,2,5,7].

## 3. Materials and Methods

### 3.1. Plant Materials

*P. grandiflorum* cv. Samidare was cultivated in the University Farm, Graduate School of Agriculture, Nagoya University, and in the Botanical Garden, Nagoya University Museum (Nagoya, Japan). The fresh petals were frozen and stored at −20 °C before use.

### 3.2. General

UV-Vis absorption spectra were recorded using a JASCO V-560 spectrometer (Jasco, Hachioji, Japan). Circular dichroism (CD) was measured using a CD J-720 spectrometer (JASCO). The spectra were obtained using a quartz cell with a path length of 10 mm. The electrospray time of flight mass spectrometry (ESI-TOF-MS) and HR-MS data were recorded using a Bruker micrOTOF-QII (ESI) spectrometer (Bruker, Billerica, MA, USA). ^1^H NMR spectra were recorded (5.0 mm i.d. tube, 5%TFA*d*-CD_3_OD) with a Bruker AVANCE III HD600 spectrometer (^1^H: 600 MHz, ^13^C: 150 MHz, Bruker, Billerica, MA, USA) and JEOL ECA-500 spectrometer (^1^H: 500 MHz, ^13^C: 125 MHz, JEOL, Akishima, Japan). ^1^H-NMR chemical shifts are reported relative to CD_2_HOD (3.31 ppm) in deuterated methanol. ^13^C NMR chemical shifts are reported relative to the NMR solvent (CD_3_OD: *δ* 49.0 ppm) as an internal reference. Analytical HPLC was performed using a JASCO HPLC system (Jasco, Hachioji, Japan) comprising two PU-1585 pumps, an HG-1580-32 mixer, a DG-1580-53 degasser, an MD-1515 detector, and a CO-1565 column oven. The system was controlled using ChromNAV ver 2 application. Reverse-phase columns (Develosil ODS-HG5, 2.0 mm i.d. × 250 mm, Nomura Chemical, Seto, Japan) were employed. Preparative HPLC was performed using a JASCO preparative HPLC system (Jasco, Hachioji, Japan) comprising an 880-PR pump, a UV-970 detector, an RC-250 recorder, and a thermostatic oven (Jr-80, TAITEC, Koshigaya, Japan). Develosil ODS-5 columns (20 mm i.d. *×* 250 mm, Nomura Chemical) and ODS-HG5 column (20 mm i.d. *×* 250 mm, Nomura Chemical) were used.

### 3.3. Extraction of Petals for HPLC Analysis

For HPLC analysis, fully opened petals (740 mg) of Chinese bellflower were frozen using liquid N_2_, followed by extraction with 3.7 mL of 3% TFA in 50% aq. acetonitrile (CH_3_CN) at room temperature for 4 h. The extract was filtered using a cartridge filter (pore size: 0.45 μm), and the filtrate was analyzed by HPLC using linear gradient elution of 10% to 37% aq. CH_3_CN solution containing 0.5% TFA for 20 min at 40 °C.

### 3.4. Purification of Anthocyanins ***1*** and ***3**–**8***

Frozen petals (1.2 kg) of Chinese bellflower, *P. grandiflorum* cv. Samidare, were extracted with 5 L of 5% HCl-methanol (CH_3_OH) overnight at room temperature. After filtration, the residue was extracted twice with 5 L of 0.1% HCl-CH_3_OH. The combined extract was concentrated under reduced pressure to approximately 1 L, and the obtained solution was washed with Et_2_O (1 L) four times. The washed aqueous solution was concentrated to 100 mL, and the condensed solution was poured into an Amberlite XAD-7 (ORGANO, Tokyo, Japan) column (80 mm i.d., 450 mm). The column was eluted with a stepwise gradient of H_2_O (5 L), 20% aq. CH_3_OH (2.8 L), and 40% aq. CH_3_OH (5 L). The 40% aq. CH_3_OH fraction was evaporated to afford crude anthocyanin (1.8 g) as a dark red mass [7]. Further purification was performed using preparative ODS-HPLC column and elution with aq. acetic acid (AcOH) and CH_3_CN containing 1% TFA to obtain pure 1 as a dark-red amorphous TFA salt (77 mg from 540 mg crude anthocyanin). To obtain minor anthocyanins, crude anthocyanin (740 mg) was purified using an ODS-HG5 column eluted with a stepwise gradient of 10% to 50% aq. CH_3_CN containing 0.5% TFA at 40 °C. For the isolation of **6** and **7**, recycled HPLC was carried out using 18% aq. CH_3_CN containing 0.5% TFA as the eluent. Pure **3** (5.7 mg), **4** (9.6 mg), **5** (6.3 mg), **6** (5.2 mg), **7** (2.0 mg), and **8** (4.1 mg) were obtained as dark-red amorphous TFA salts.

**5**: UV-Vis: λ_max_ (ε) (0.1% HCl-CH_3_OH) 285 nm (25,700), 548 nm (19,200); CD: λ (∆ε) 548 nm (−9.95), 360 nm (+0.57), 338 nm (−2.06), 317 nm (+3.08), 297 nm (−2.38), 278 nm (+10.67), 252 nm (+2.94).

**6**: UV-Vis: λ_max_ (0.1% HCl-CH_3_OH) 285 nm (12,700), 549 nm (8600); CD: λ (∆ε) 547 nm (−4.44), 352 nm (+0.37), 315 nm (2.06), 296 nm (−1.18), 278 nm (+4.76), 252 nm (+1.48).

**7**: UV-Vis: λ_max_ (0.1% HCl-CH_3_OH) 285 nm (18,300), 548 nm (11,100); CD: λ (∆ε) 549 nm (−6.16), 354 nm (+0.73), 331 nm (−1.72), 312 nm (+1.86), 296 nm (−1.02), 277 nm (+8.55).

**8**: UV-Vis: λ_max_ (0.1% HCl-CH_3_OH) 286 nm (26,800), 547 nm (22,100); CD: λ (∆ε) 546 nm (−8.28), 356 nm (+1.56), 322 nm (+4.99), 297 nm (−3.81), 279 nm (+7.46), 250 nm (+1.81).

### 3.5. Alkaline Hydrolysis of ***1***

Alkaline hydrolysis of **1** was performed according to the method described in a previous report [7]. Crude 1 (30 mg; >70% purity) was dissolved in CH_3_OH (0.5 mL), and argon atmosphere was used. The solution was cooled to 0 °C, after which 1% aq. NaOH (0.5 mL) was added, and the mixture was allowed to stand for 15 min. After the addition of aq. HCl (18%, 100 μL), the mixture was concentrated under reduced pressure and purified using preparative HPLC (Develosil ODS-HG5). Pure **2** (1.6 mg) was obtained as a dark-red TFA salt.

### 3.6. Stability of Anthocyanins

Each anthocyanin (**1**–**8**) was dissolved in water at a concentration of 5 mM, and diluted with phosphate buffer (200 mM, pH 6.0) to a concentration of 50 μM. Thereafter, the solution was poured into a quartz cell (path length 10 mm) and the UV-Vis spectrum (200–800 nm) was recorded. The solutions were stored in the dark at 25 °C, and the UV-Vis measurements were repeated. Stability was monitored by examining the decrease in absorbance at λvis_max_.

## 4. Conclusions

In conclusion, seven minor anthocyanins (**2**–**8**) in addition to platyconin (1) were identified in the bluish-purple petals of the Chinese bellflower, *P. grandiflorum*. These anthocyanins were purified, and their structures were elucidated using various analytical techniques. Three of these compounds were new diacylanthocyanins containing a delphinidin chromophore with a straight glucosyl-acyl-glucosl-acyl chain at the 7 position. In a buffer solution of pH 6.0, diacylanthocyanins (**1**, **5**–**8**) exhibited a bluish-purple color with λ_max_ values of approximately 570 and 617 nm. Although the blue hues were slightly different in each sample, all diacylanthocyanins showed high stability, retaining >80% of the color for a week. Intramolecular stacking between the delphinidin chromophore and acyl moieties could be suggested. The variation in the acyl substitution order at 7-OH of the delphinidin nucleus indicates the redundancy of biosynthesis in terms of acylation.

## Figures and Tables

**Table 1:** HR-MS data of diacylated anthocyanins **1** and **5**–**8**.

	Molecular Ion Peak (Obs.)	Calcd.	Molecular Formula
**1**	1421.3825	1421.3826	C_63_H_73_O_37_
**5**	1275.3246	1275.3240	C_57_H_63_O_33_
**6**	1405.3876	1405.3879	C_63_H_73_O_36_
**7**	1405.3876	1405.3864	C_63_H_73_O_36_
**8**	1259.3297	1259.3291	C_57_H_63_O_32_

**Table 2:** Assignment of ^1^H and ^13^C NMR spectra of dianthocyanins, **1**, **5**–**8** in 5% TFAd-CD_3_OD.

		1				5				6				7				8			
		^1^H			^13^C	^1^H			^13^C	^1^H			^13^C	^1^H			^13^C	^1^H			^13^C
		*δ* (ppm)	multi.	*J* (Hz)	*δ* (ppm)	*δ* (ppm)	multi.	*J* (Hz)	*δ* (ppm)	*δ* (ppm)	multi.	*J* (Hz)	*δ* (ppm)	*δ* (ppm)	multi.	*J* (Hz)	*δ* (ppm)	*δ* (ppm)	multi.	*J* (Hz)	*δ* (ppm)
Del	2				162.2				162.4				162.4				162.1				162.9
	3				145.9				145.8				145.9				145.9				145.9
	4	8.60	s		131.7	8.68	s		131.8	8.56	s		131.7	8.61	s		131.9	8.70	s		131.8
	5				156.0				156.2				155.9				156.0				159.4
	6	6.77	d	1.5	104.0	6.86	d	2.5	104.1	6.72	brs		104.3	6.75	br. d	1.5	1041.0	6.77	d	1.5	103.1
	7				165.5				165.7				165.5				165.6				165.6
	8	6.98	brd	1.5	93.1	6.70	br.d	2.5	93.1	7.04	brs		93.1	7.00	br. s		93.0	7.00	br. d	1.5	93.5
	9				154.5				154.5				154.5				154.6				154.7
	10				112.2				112.2				112.1				112.3				112.2
	1’				118.5				118.6				118.4				118.7				118.4
	2’	7.81	brs		112.2	7.86	d	2.5	111.8	7.85	brs		111.9	7.88	br. s		111.9	7.76	br. s		112.1
	3’				145.0				144.8				144.9				144.8				145.9
	4’				146.3				146.4				147.1				146.4				145.9
	5’				145.0				144.8				144.9				144.8				145.0
	6’	7.81	brs		112.2	8.29	dd	2.5, 8.5	112.2	7.85	brs		111.9	7.88	br. s		111.9	7.76	br. s		112.1
G1	1	5.29	d	7.5	101.7	5.31	d	6.5	104.1	5.24	d	7.8	101.9	5.32	br.d	7.5	101.5	5.24	d	7.5	102.0
	2	3.85	dd	7.5, 9.5	73.1	3.89	dd	6.5, 8.0	73.2	3.80	dd	7.8, 9.5	73.1	3.81	dd	7.5, 9.5	73.1	3.89	dd	7.5, 9.5	73.1
	3	3.60	dd	8.0, 9.5	76.4	3.61	dd	8.0, 9.0	76.7	3.57	t	9.5	76.4	3.61	t	9.5	75.8	3.60	dd	8.0, 9.5	76.4
	4	3.54	t	9.5	69.6	3.59	t	9.0	69.8	3.49	t	9.5	70.0	3.55	t	9.5	69.6	3.49	t	9.5	69.9
	5	3.79	m		76.5	3.70	ddd	2.5, 5.5, 9.0	77.8	3.78	m		76.4	3.80	m		76.4	3.80	br. dt	1.5, 9.5	76.6
	6a	4.26	m		66.4	4.13	dd	2.5, 12.0	61.2	4.27	brd	10.5	66.7	4.28	dd	9.5, 12.0	66.4	4.20	br. dd	1.5, 12.0	66.6
	6b	3.79	m			3.98	dd	5.5, 12.0		3.80	m			3.80	m			3.73	br. d	9.5,12.0	
G2	1	5.19	d	7.5	100.4	5.18	d	7.5	100.3	5.21	d	7.8	100.0	5.20	br. d	7.5	100.4	4.92	d	7.5	99.9
	2	3.73	dd	7.5, 9.5	72.8	3.72	dd	7.5, 9.0	72.8	3.72	dd	7.8, 9.5	72.8	3.74	br. dd	7.5, 9.5	72.8	3.67	dd	7.5, 9.5	76.2
	3	3.60	t	9.5	75.8	3.58	t	9.0	76.3	3.59	t	9.5	76.3	3.61	br. t	9.5	76.3	3.59	t	9.5	76.5
	4	3.49	t	9.5	70.7	3.48	t	9.0	69.7	3.47	t	9.5	71.8	3.49	br. t	9.5	70.7	3.46	t	9.5	70.7
	5	3.99	ddd	2.0, 9.5, 10.0	74.6	3.95	ddd	2.5, 5.5, 9.0	74.6	4.00	m		74.7	4.03	br. ddd	2.0, 9.5, 10.0	74.6	3.91	br. m		74.6
	6a	4.73	dd	10.0, 12.0	64.4	4.74	dd	2.5, 12.0	64.4	4.75	d	12.0	64.4	4.67	br. dd	10.0, 12.0	64.6	4.26	dd	10.0, 12.0	63.9
	6b	4.37	dd	2.0, 12.0		4.36	dd	5.5, 12.0		4.37	dd	9.0, 12,0		4.42	br. dd	2.0, 12.0		4.83	dd	2.0, 12.0	
G3	1	4.92	d	7.5	100.5	4.96	d	7.5	100.4	4.93	d	7.5	100.5	5.01	br. d	7.5	99.3	4.92	d	7.5	100.7
	2	3.61	dd	7.5, 9.5	73.2	3.61	dd	7.5, 9.0	73.0	3.61	dd	7.5, 9.5	73.2	3.56	br. dd	7.5, 9.5	73.0	3.61	dd	7.5, 9.5	73.2
	3	3.70	t	9.5	76.1	3.69	t	9.0	76.2	3.71	t	9.5	76.2	3.70	br. t	9.5	76.6	3.70	t	9.5	73.0
	4	3.38	t	9.5	71.6	3.39	t	9.0	71.4	3.97	t	9.5	71.6	3.37	br. t	9.5	71.6	3.38	t	9.5	71.2
	5	3.80	m		73.5	4.00	ddd	2.5, 5.5, 9.0	73.3	3.97	m		73.6	4.00	br. m		73.3	3.93	m		73.9
	6a	4.73	brd	2.5, 12.0	64.4	4.80	dd	2.5, 12.0	64.5	4.75	brd	11.0	64.3	4.77	br. dd	2.5, 12.0	64.5	4.71	br. d	2.5, 12.0	63.7
	6b	3.80	m			3.95	dd	5.5, 12.0		4.02	brd	11.0		3.91	br. m			4.38	dd	9.0, 12.0	
G4	1	4.60	d	7.5	101.4	4.53	d	7.5	101.6	4.73	d	7.8	99.7	4.57	br. d	7.5	101.0				
	2	3.47	dd	7.5, 9.5	73.3	3.49	dd	7.5, 9.0	73.3	3.44	dd	7.8, 9.5	72.7	3.46	br. dd	7.5, 9.5	73.2				
	3	3.60	t	9.5	76.3	3.60	t	9	75.8	3.56	t	9.5	76.5	3.61	br. t	9.5	76.5				
	4	3.46	t	9.5	69.8	3.48	t	9	70.7	3.47	m		70.0	3.50	br. t	9.5	69.8				
	5	3.50	m		76.5	3.43	ddd	2.5, 5.5, 9.0	76.4	3.47	m		76.3	3.48	br. m		76.3				
	6a	3.85	dd	2.0, 12.0	60.8	3.80	dd	2.5,12.5	60.6	3.89	dd	1.5, 11.0	61.0	3.83	br. dd	2.0, 12.0	60.7				
	6b	3.75	dd	4.5, 12.0		3.75	dd	5.5, 12.5		3.75	brd	11.0		3.77	br. dd	4.5, 12.0					
Rha	1	4.89	brd	1.0	101.7					4.86	brs		100.9	4.87	br. s		101.1	4.81	br. d	1.0	101.7
	2	4.09	dd	1.0, 3.5	70.3					4.05	brdd	1.5, 3.0	70.4	4.11	br. d	3.5	70.3	4.01	dd	1.0, 3.5	70.3
	3	3.72	dd	3.5, 9.5	71.2					3.75	dd	3.0, 9.5	71.2	3.72	dd	3.5, 9.5	71.2	3.74	dd	3.5, 9.5	71.2
	4	3.40	t	9.5	72.5					3.43	t	9.5	69.8	3.40	t	9.5	72.5	3.40	t	9.5	72.5
	5	3.67	dt	6.0, 9.5	70.3					3.65	dt	6.5, 9.5	68.6	3.66	m		68.6	3.65	dt	6.0, 9.5	68.5
	6	1.26	d	6.0	16.5					1.28	d	6.5	16.6	1.25	dd	6.0	16.5	1.25	d	6.0	16.5
acyl1	1				128.7				128.6				128.6				127.8				128.7
	2	6.80	d	2.5	116.9	6.88	d	2.5	116.9	6.87	d	1.5	116.5	7.33	br. d	8.5	129.4	6.80	d	2.5	115.9
	3				146.3				146.4				146.5	6.82	br. d	8.5	115.9				146.4
	4				147.1				147.2				147.1				159.2				147.1
	5	6.72	d	8.5	115.1	6.79	d	8.5	115.0	6.75	d	8.5	115.3	6.82	br. d	8.5	119.9	6.73	d	8.5	115.3
	6	6.67	dd	2.5, 8.5	118.4	6.86	dd	2.5, 8.5	118.0	6.73	dd	1.5, 8.5	118.4	7.33	br. d	8.5	129.4	6.51	dd	2.5, 8.5	119.1
	α	5.84	d	15.5	115.2	6.50	d	15.5	118.5	6.45	d	16.0	115.0	6.55	d	16.0	114.7	6.28	d	15.5	115.3
	β	7.13	d	15.5	144.7	7.55	d	15.5	145.2	7.53	d	16.0	145.3	7.64	d	16.0	144.9	7.38	d	15.5	144.9
	CO				167.5				167.4				167.3				167.7				167.3
acyl2	1				129.1				128.7				128.0				129.0				126.0
	2	6.64	d	2.5	113.6	6.91	d	2.5	113.6	7.09	d	8.5	128.6	6.78	br. s		113.5	6.80	d	2.5	113.5
	3				146.8				145.8	6.72	d	8.5	116.4				146.6				146.2
	4				146.7				146.8				158.8				146.8				147.8
	5	6.63	d	8.5	115.8	6.68	d	8.5	115.6	6.72	d	8.5	116.4	6.55	br. d	8.5	115.3	6.49	d	8.5	115.0
	6	6.57	dd	2.5, 8.5	120.4	6.47	dd	2.5, 8.5	121.0	7.09	d	8.5	128.6	6.32	br. d	8.5	120.4	6.54	dd	2.5, 8.5	121.2
	α	5.77	d	15.5	116.9	6.16	d	15.5	116.9	6.14	d	16.0	116.5	6.12	d	16.0	117.0	6.09	d	15.5	114.1
	β	6.99	d	15.5	143.2	7.01	d	15.5	143.4	7.12	d	16.0	143.0	6.90	br. d	16.0	143.0	7.21	d	15.5	144.8
	CO				166.8				167.0				166.9				166.9				167.3

## Data Availability

Data available on request to the correspondence author.

## Supplementary Materials

The following are available online at https://www.mdpi.com/article/10.3390/ijms22084044/s1.
