# Supplementary material for: Polyacylated Anthocyanins in Bluish-Purple Petals of Chinese Bellflower, Platycodon grandiflorum
Source: Int J Mol Sci. 2021 Apr 14;22(8):4044. doi: 10.3390/ijms22084044 (PMC8070928; doi:10.3390/ijms22084044)
Supplement: Supplementary file 1 [file ijms-22-04044-s001.pdf]

Supporting Information

Polyacylated anthocyanins in bluish-purple petals of Chinese bellflower, *Platycodon grandiflorum*

Tadao Kondo [1], Seiji Hagihara [1], Yoshiaki Takaya [2] and Kumi Yoshida [1,*]

[1] Graduate School of Informatics, Nagoya University, Chikusa, Nagoya 464-8601
kondot@info.human.nagoya-u.ac.jp, hagihara.seiji@j.mbox.nagoya-u.ac.jp
[2] Faculty of Pharmacy, Meijo University, 150 Yagotoyama, Tenpaku, Nagoya 468-8503, Japan
ytakaya@meijo-u.ac.jp
* Correspondence: yoshidak@i.nagoya-u.ac.jp; Tel.: +81-052-789-5638

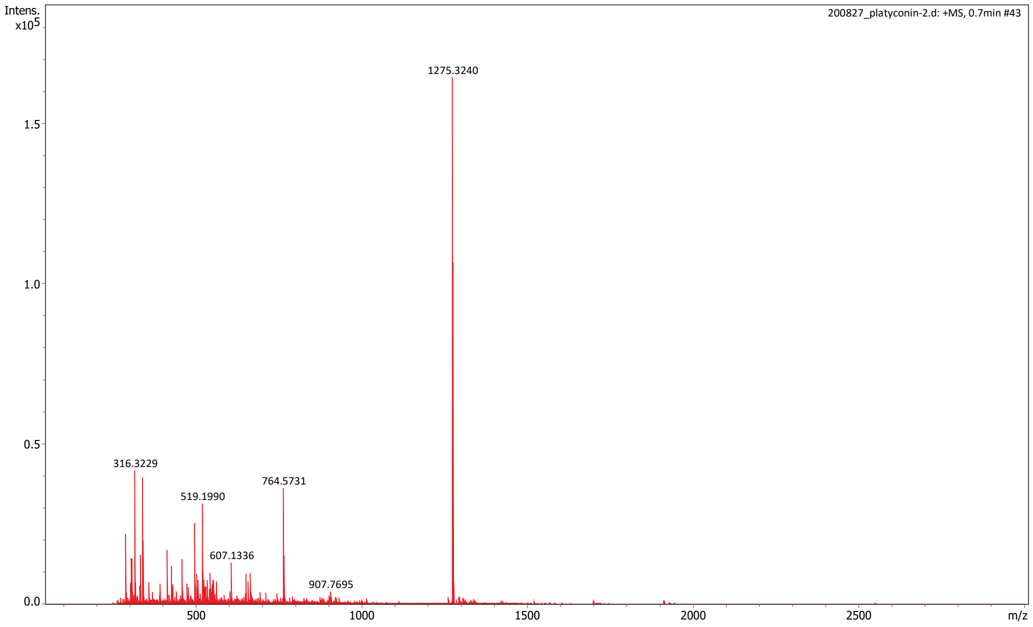

Figure S1. HR-MS of **5**.

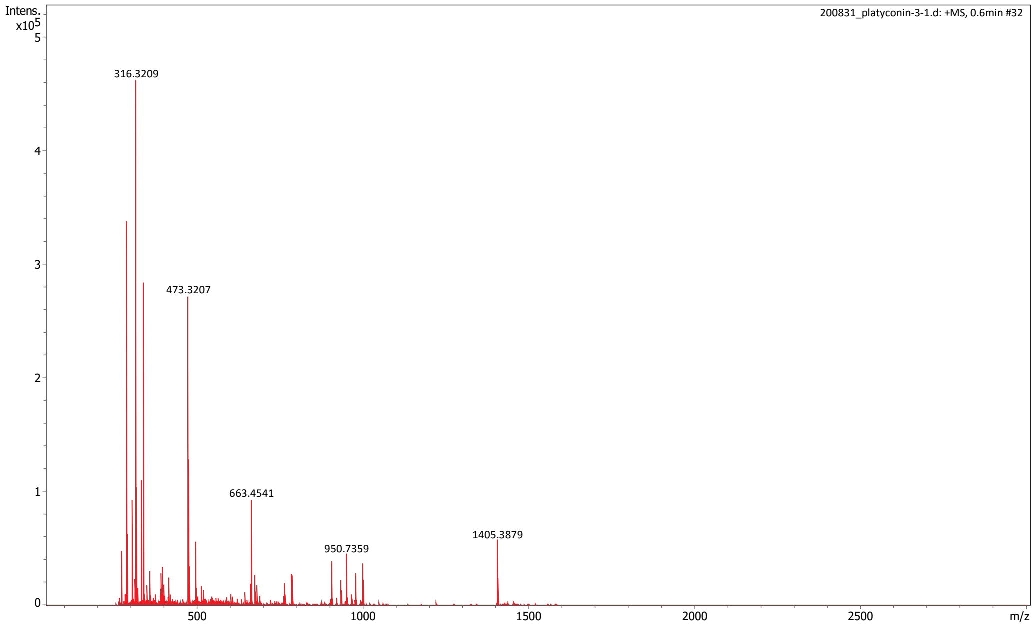

Figure S2. HR-MS of **6**.

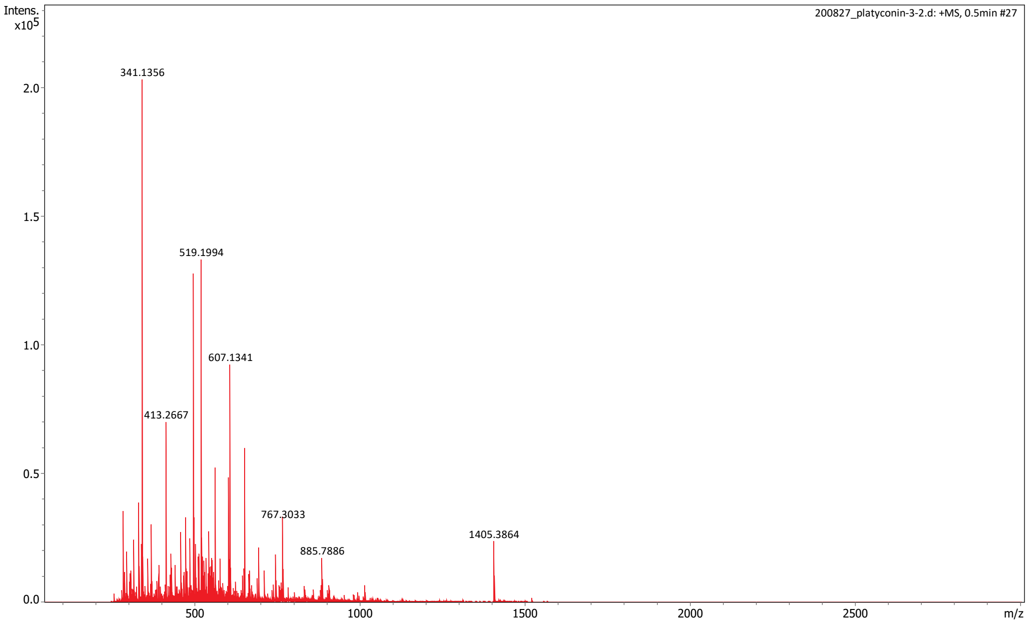

Figure S3. HR-MS of **7**.

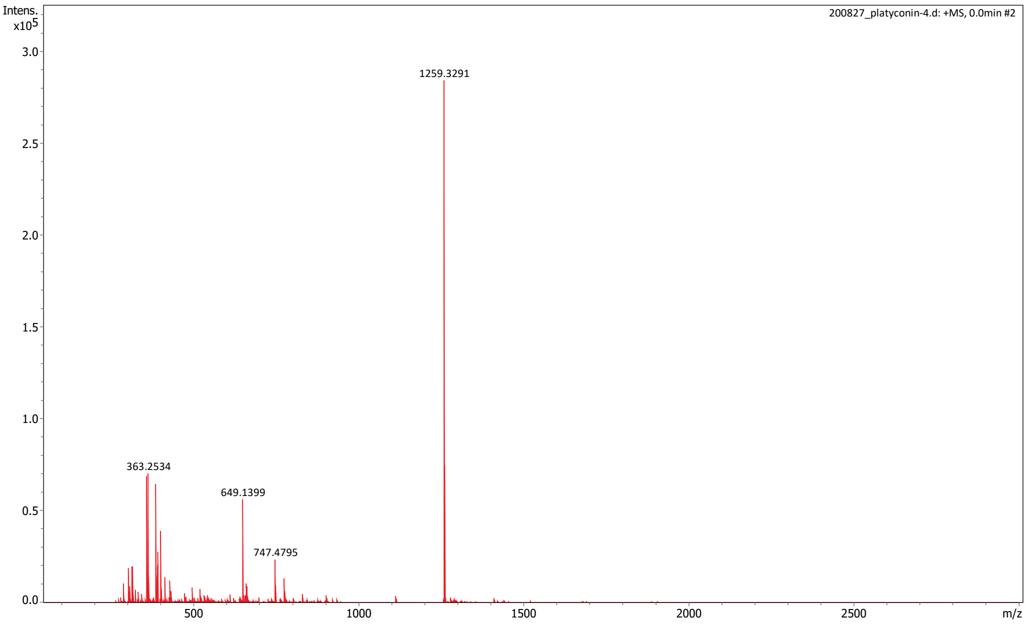

Figure S4. HR-MS of **8**.

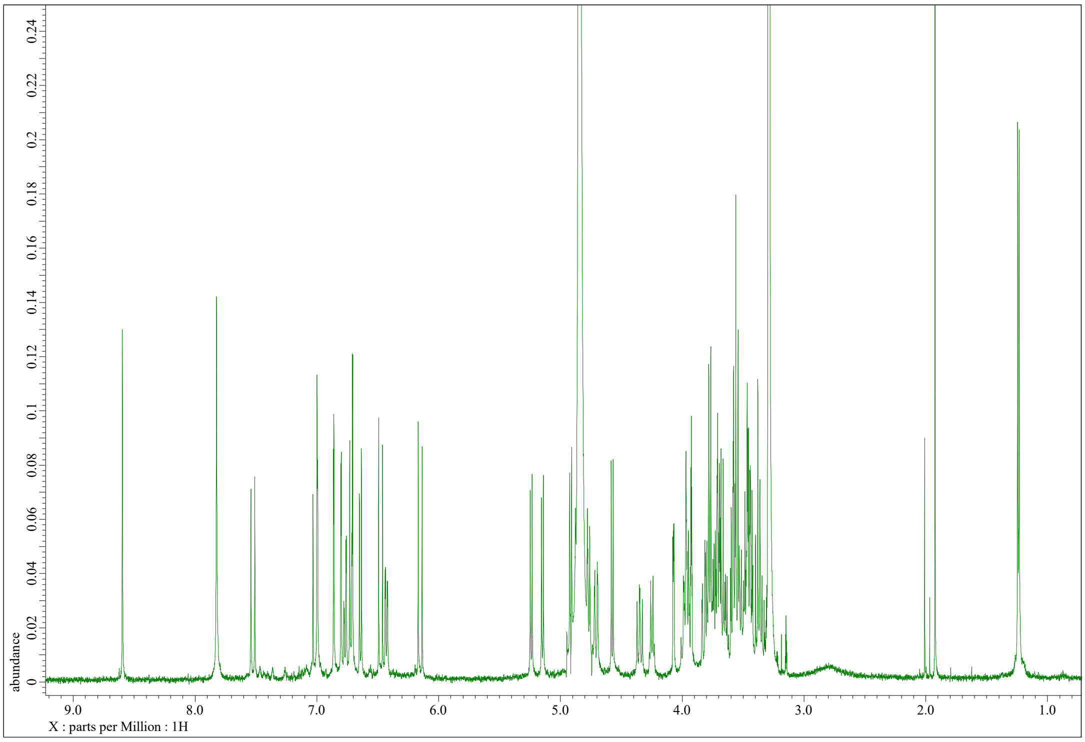

Figure S5. ^{1}H NMR spectrum of **1** (^{1}H: 500 MHz, 5% TFA*d*-CD_3OD, rt).

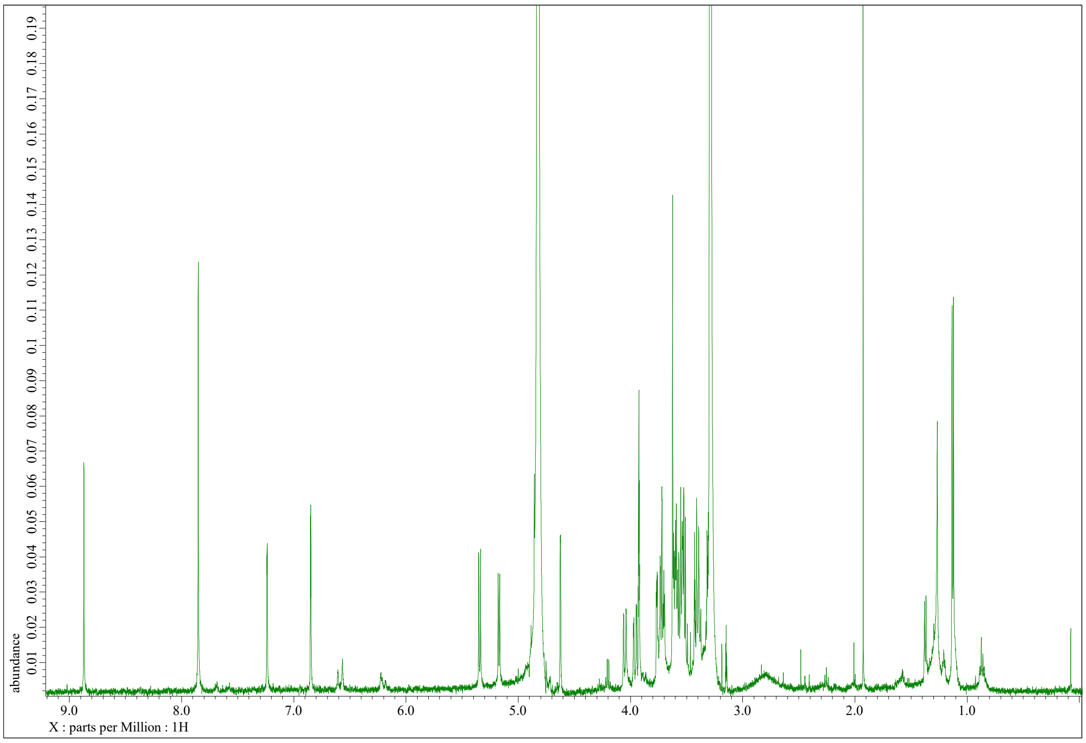

Figure S6. ^{1}H NMR spectrum of **2** (^{1}H: 500 MHz, 5% TFA*d*-CD_3OD, rt).

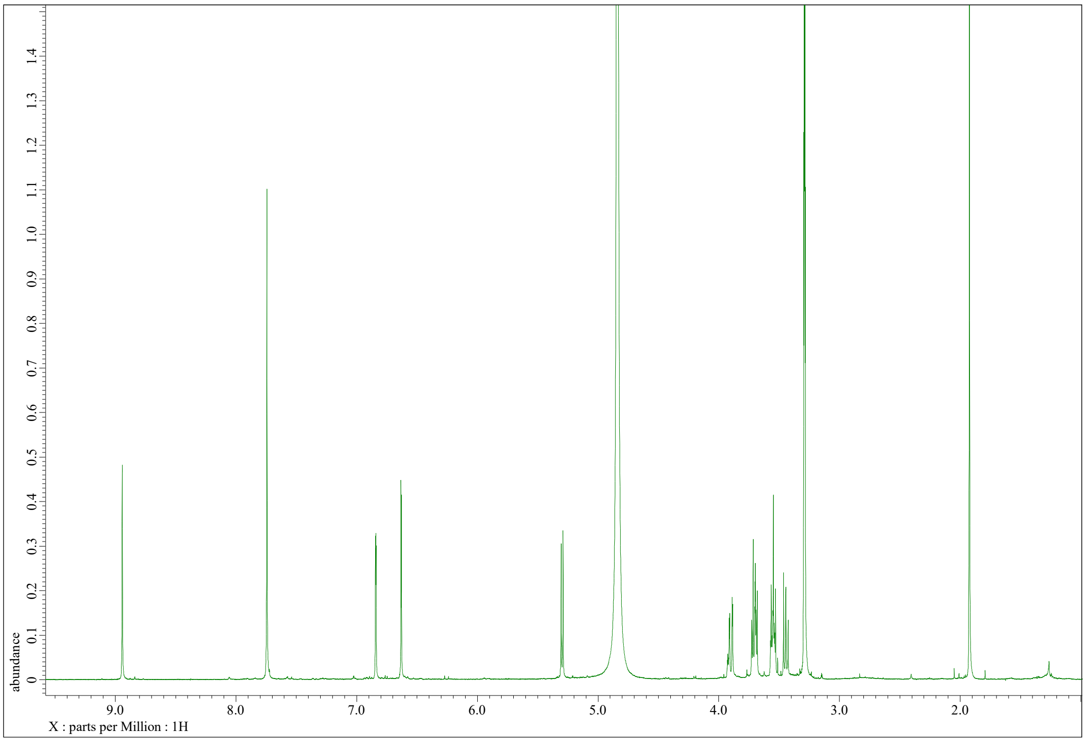

Figure S7. ^{1}H NMR spectrum of **3** (^{1}H: 500 MHz, 5% TFA*d*-CD_3OD, rt).

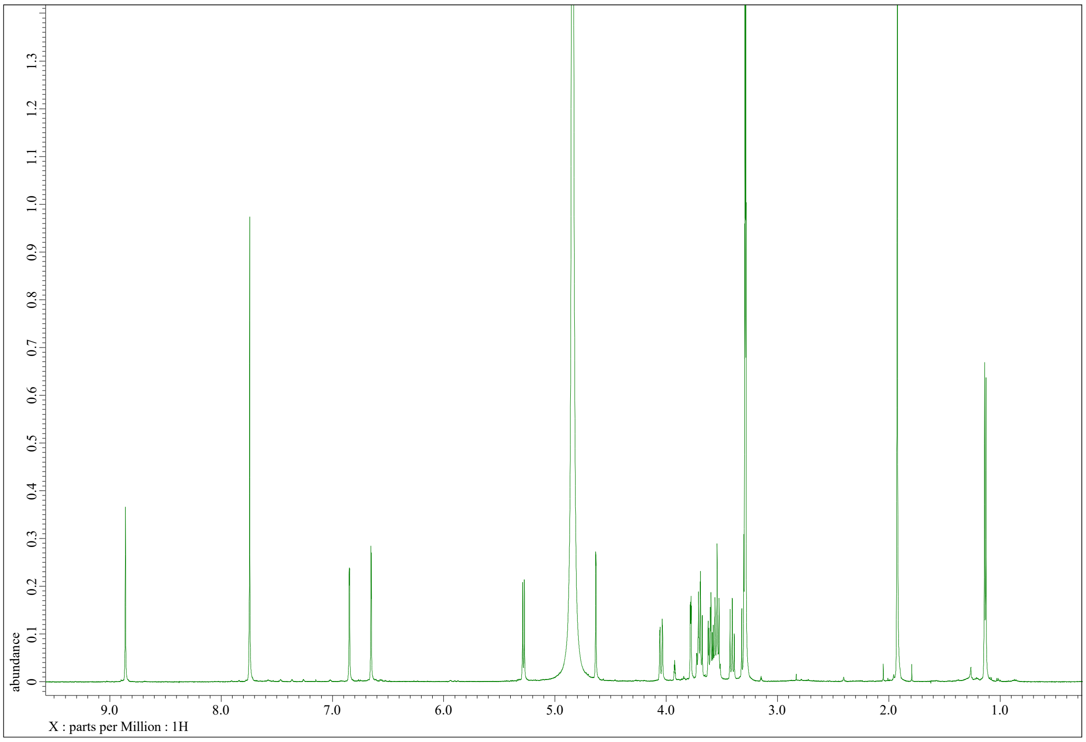

Figure S8. ^{1}H NMR spectrum of **4** (^{1}H: 500 MHz, 5% TFA*d*-CD_3OD, rt).

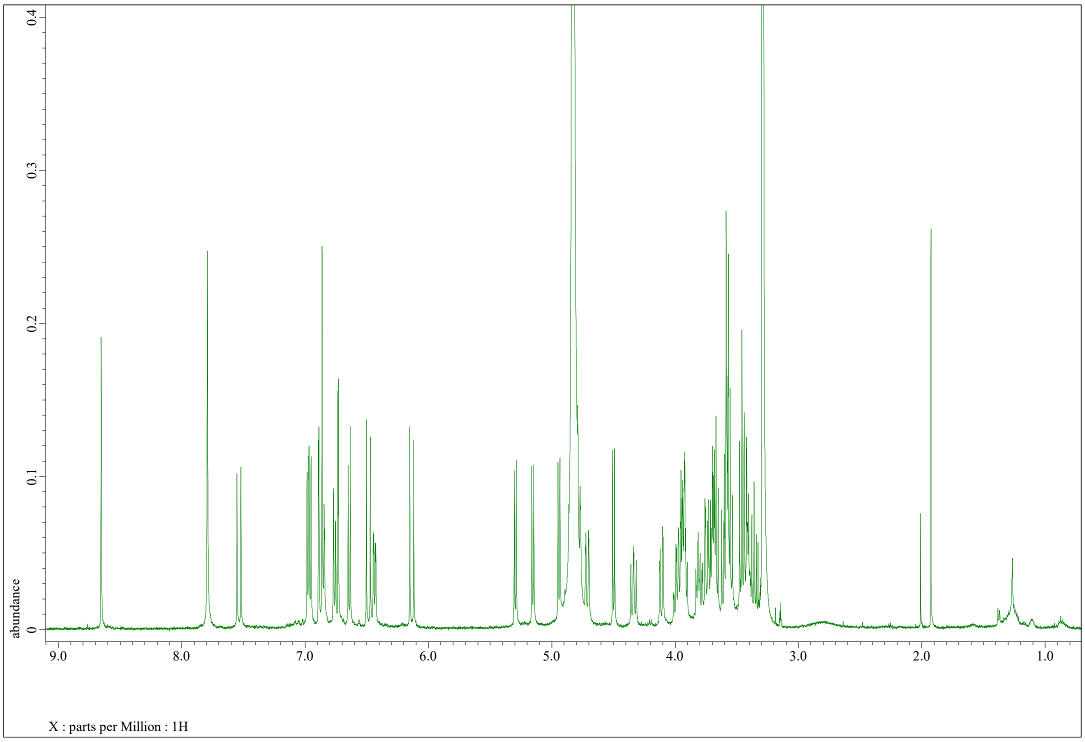

Figure S9. ^{1}H NMR spectrum of **5** (^{1}H: 500 MHz, 5% TFA*d*-CD_3OD, rt).

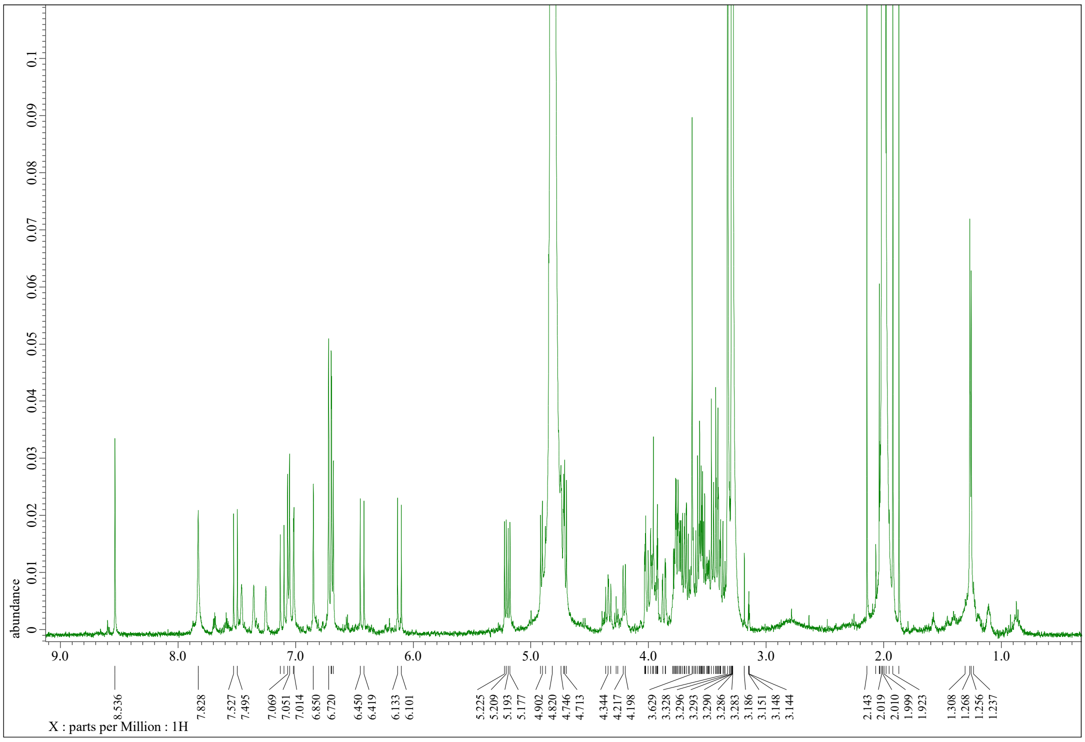

Figure S10. ^{1}H NMR spectrum of **6** (^{1}H: 500 MHz, 5% TFA*d*-CD_3OD, rt).

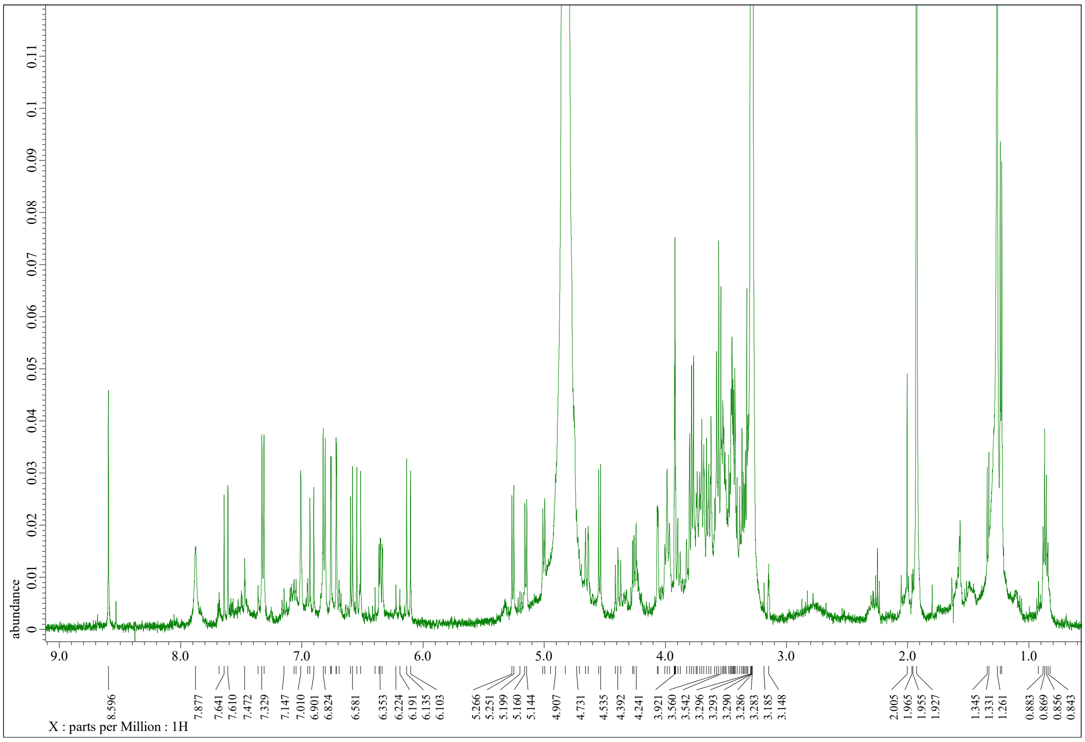

Figure S11. ^{1}H NMR spectrum of **7** (^{1}H: 500 MHz, 5% TFA*d*-CD3OD, rt).

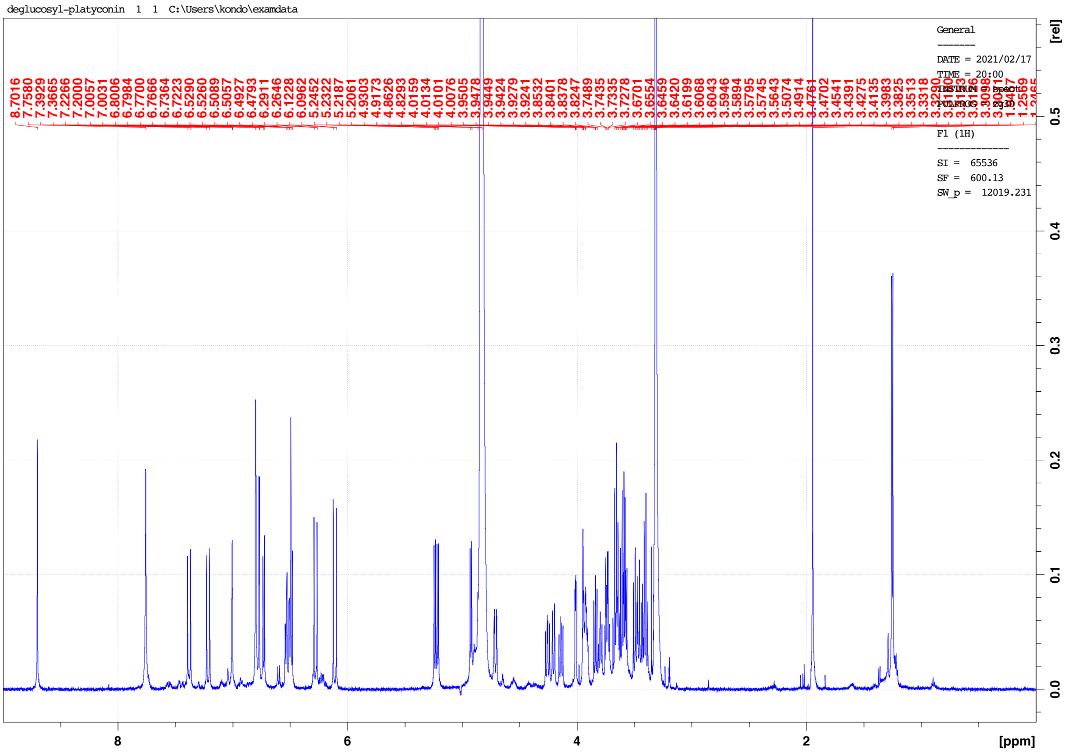

Figure S12. ^{1}H NMR spectrum of **8** (^{1}H: 500 MHz, 5% TFA*d*-CD3OD, rt).

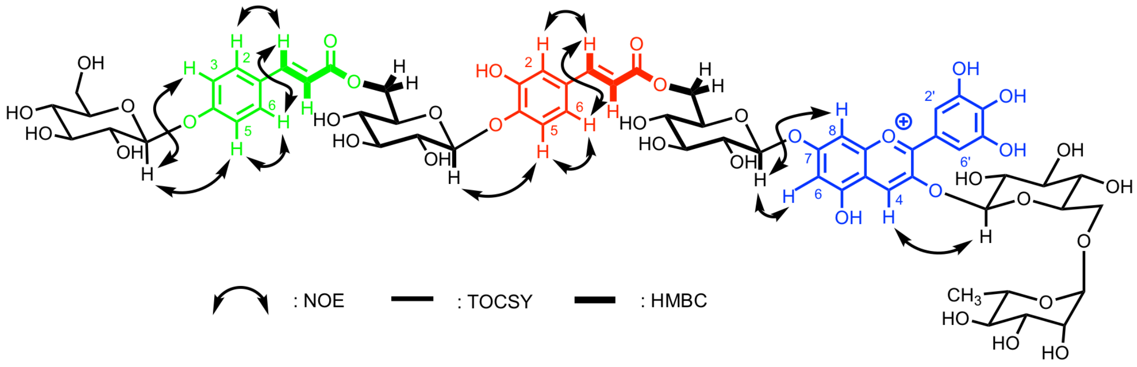

Figure S13. Strategy of structural determination of **6**. Attaching positions of glucose were determined by NOE correlations indicated by double-edged arrow, the signals of sugar were assigned by using 1D-TOCSY correlations, and the attachment between sugar and acyl moiety was determined by using HMBC correlation. The same procedure was applied to determine the structure of **7** and other diacylated anthocyanins.
